# Metabolic syndrome and metastatic prostate cancer correlation study, a real-world study in a prostate cancer clinical research center, Xinjiang, China

**DOI:** 10.3389/fendo.2022.1090763

**Published:** 2022-12-06

**Authors:** Hengqing An, Dongsheng Ma, Yujie Mei, Lulu Wang, Abudukeyoumu Maimaitiyiming, Tao Zhuo, Renaguli Aihaiti, Ke Bu, Xin Huang, Kaige Zhang, Miao Yao, Chenyang Ling, Weizun Li, Ning Tao

**Affiliations:** ^1^ The First Affiliated Hospital, Xinjiang Medical University, Urumqi, China; ^2^ Department of Urology, The First Affiliated Hospital of Xinjiang Medical University, Urumqi, China; ^3^ Xinjiang Clinical Research Center of Urogenital Diseases, Urumqi, China; ^4^ College of Public Health, Xinjiang Medical University, Urumqi, China; ^5^ Department of Epidemiology and Health Statistics, College of Public Health, Xinjiang Medical University, Urumqi, China

**Keywords:** metabolic syndrome, metastatic prostate cancer, central obesity, insulin resistance, androgen deprivation therapy, novel endocrine therapy

## Abstract

**Objective:**

The aim of this study was to investigate the relevance of metabolic syndrome (MetS) and metabolic scores to the occurrence, progression and prognosis of metastatic prostate cancer (mPCA), assessing the definition of the variables of metabolic syndrome, and the potential mechanisms of MetS and mPCA.

**Methods:**

Data were obtained from the database of prostate cancer follow-up at the Urology Centre of the First Affiliated Hospital of Xinjiang Medical University (N=1303). After screening by inclusion and exclusion criteria, clinical data of 190 patients diagnosed with mPCA by pathology and imaging from January 2010 to August 2021 were finally included, including 111 cases in the MetS group and 79 cases in the Non-MetS group.

**Results:**

The MetS group was higher than the Non-MetS group: T stage, Gleasson score, initial PSA, tumor load, PSA after 7 months of ADT (*P*<0.05),with a shorter time to progression to CRPC stage(*P*<0.05)[where the time to progression to CRPC was relatively shorter in the high metabolic score subgroup of the MetS group than in the low subgroup (*P*<0.05)].Median survival time was significantly shorter in the MetS group than in the Non-MetS group (*P*<0.05),and there was a correlation with metabolic score, with the higher metabolic score subgroup having a lower survival time than the lower metabolic score subgroup (*P*<0.05).

**Conclusion:**

Those with mPCA combined with MetS had lower PSA remission rates, more aggressive tumors, shorter time to progression to CRPC and shorter median survival times than those with mPCA without MetS.Tumour progression and metabolic score showed a positive correlation, predicting that MetS may promote the progression of mPCA, suggesting that MetS may be a risk factor affecting the prognosis of mPCA.

## Introduction

Metabolic syndrome (MetS) is a group of clinical syndromes characterised by the aggregation of multiple disease states, including abdominal obesity, persistent hypertension, dyslipidaemia, abnormalities of glucose metabolism (1).MetS can significantly affect the occurrence, development and prognosis of other related diseases, increase the incidence of cardiovascular accidents and cancer, and result in increased hospitalization, surgical complications and mortality compared to non-MetS patients. The National Cholesterol Education Program Adult Treatment Panel III (NCEP ATP III) defines MetS in a way that is widely accepted by the academic community, and considers that patients who meet three of the five criteria of abdominal obesity, hyperlipidaemia, hypertension, elevated fasting glucose levels and decreased HDL cholesterol levels are diagnosed with MetS. In 2017, the Chinese Guidelines for the Prevention and Treatment of Type 2 Diabetes published in China revised and published the diagnostic criteria for MetS in China based on this diagnostic criteria, with reference to the definition of abdominal obesity and hyperglycaemia in China. The MetS was first proposed as a risk-related compound factor in 2004, and middle-aged men with metabolic syndrome are more likely to develop prostate cancer (PCA) (2). Given the presence of complex hormonal and metabolic changes in MetS, and the prostate as a specific endocrine and reproductive organ in men, it is predicted that there may be a relationship between MetS and PCA (3).There are differences between populations of different ethnicities and regions. The prevalence of MetS in Latin American countries is 15.5% (23.1% in men and 12.2% in women) (4). The prevalence of MetS in European and American countries is 24.3% (23.9% in men and 24.6% in women) (5). In China, recent surveys in different age and regional populations have found that the prevalence of MetS varies from approximately 3.6 to 50.1%, with significant gender and regional differences (6–8). The relationship between MetS and PCA is still not fully established. However, studies that use rigorous and uniform criteria to define the metabolic syndrome in homogeneous ethnic groups are necessary to further elucidate the link between the metabolic syndrome and prostate cancer outcomes (9).No epidemiological studies related to high-quality MetS have been reported in Xinjiang, China, but the high-fat dietary structure characteristics of Xinjiang may lead to a higher prevalence than the Chinese average, which is also corroborated by the high prevalence characteristics of diabetes, hypertension, hyperlipidemia and other related diseases in Xinjiang. As people's material life has become richer in recent years, unhealthy and irregular diet and lifestyle habits have increased the incidence of MetS year by year, and the risk of cardiovascular and cerebrovascular accidents has gradually emerged. Metabolic syndrome and cardiovascular disease are closely related and there is a significant dose-response relationship between the components of MetS and the risk of cardiovascular disease (10). Metabolism and cancer are closely related and there is a correlation between many solid tumors such as endometrial, colorectal, gastric, liver, bladder and prostate cancers (11–13). Metabolic dysfunction is associated with the risk and mortality of colorectal, pancreatic, postmenopausal breast and bladder cancers (14). PCA is currently the most prevalent malignancy of the male reproductive system worldwide, with the 2nd and 5th highest incidence and mortality rates of malignancies in men, respectively (15). Due to its high prevalence in middle-aged and elderly men and its insidious onset, patients are often in the middle to advanced stages at the time of first presentation, with metastatic prostate cancer (mPCA) being an important stage of disease that severely affects patient prognosis. In the European and American populations, mPCA accounts for only 5%-6% of new diagnosed PCA, with an overall 5-year survival rate of approximately 30% (16).The MetS component is associated with an age-specific increase in the incidence of PCA, and a history of MetS is associated with a high prevalence of PCA (17, 18).The prevalence of MetS may be higher in Xinjiang, China, than in other parts of China due to dietary habits and lifestyle.The prevalence of prostate cancer is higher in Xinjiang, China, but the treatment situation is not optimistic. While previous literature aimed to explore the relationship between MetS and the risk of PCA in different populations, this study focused on the correlation between MetS and the occurrence, progression and prognosis of mPCA in Xinjiang, China.It is expected to inform the development of scientific and effective public health policies and clinical treatment protocols.

## Materials and methods

### Data sources and descriptions

**Figure 1:**
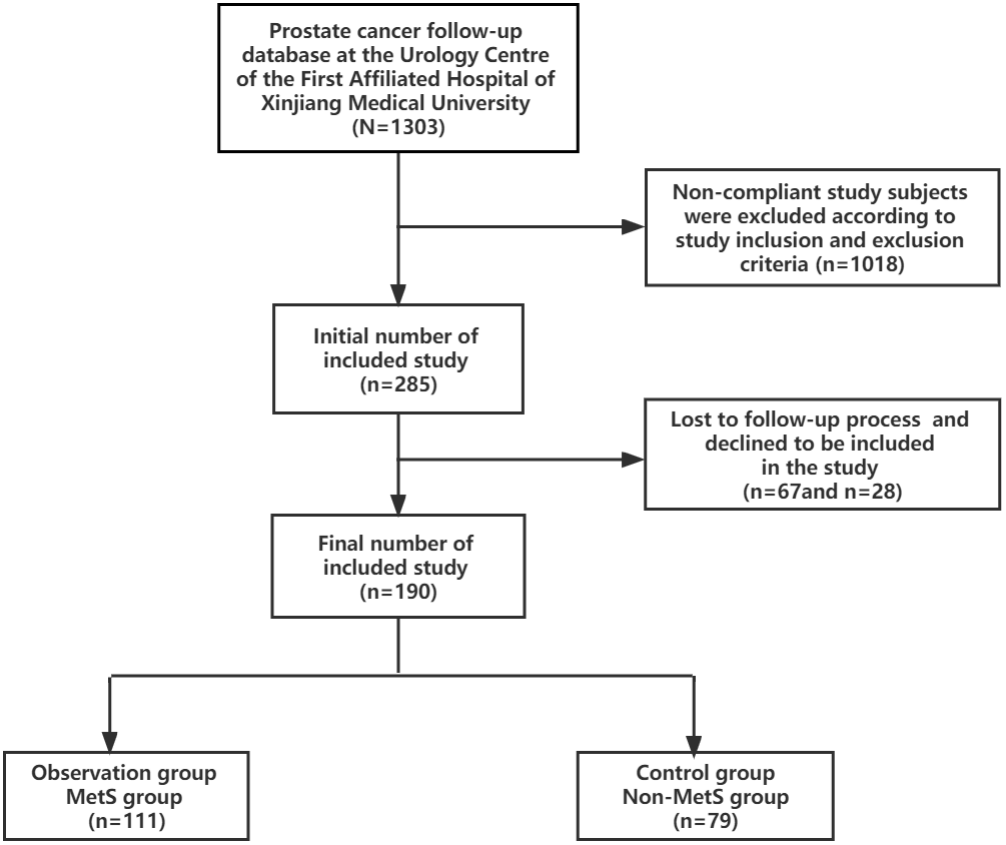
The process for study participants.

The study collected clinical data of 1303 prostate cancer patients from January 2010 to August 2021 from the prostate cancer follow-up database of the Urology Center of the First Affiliated Hospital of Xinjiang Medical University. 1018 cases were excluded according to the inclusion and exclusion criteria, 67 cases were lost in the follow-up process and 28 cases refused to be included in the study. 190 cases were finally screened and included in the study, all of which were diagnosed with mPCA by pathology and imaging, including 111 cases in the MetS group and 79 cases in the Non-MetS group.According to the clinical study design requirements, the group of mPCA patients with combined MetS was set up as the observation group and the group of mPCA patients with Non-MetS was set up as the control group. See **Figure 1**.

#### Biochemical indicators

(1) Endocrine-related indicators

Glycerin trilaurate (TG), high density lipoprotein cholesterol (HDL), fasting blood-glucose (FBG).

(2) Prostate cancer tumor-related indicators

Prostate specific antigen (PSA), serum testosterone (TE), Gleason score, tumor staging.

#### Blood pressure

Because of its fluctuating nature and the transient rise in blood pressure that can be caused by emotional or physical activity, is diagnosed when the patient's blood pressure is elevated on at least two different days at rest. If the patient has a clear previous history of hypertension, this may also be considered to meet the criteria for MetS iv.

#### Blood glucose and lipid levels

Blood was collected from the elbow vein in the morning on an empty stomach or during an 8-hour fast, and FBG and TG levels were measured.

#### Body mass index

BMI= Body weight divided by height squared(kg/m^2^)

#### Measurement of serum PSA and testosterone levels

Serum PSA and testosterone levels were measured by electrochemiluminescence in the early morning on an empty stomach, and the maximum values of PSA were determined by the department of urology of the First Affiliated Hospital of Xinjiang Medical University or the external medical institutions.

#### Gleason pathology score

The highest total Gleason score in the normative standard pathology report after the puncture is used.

#### TNM staging of tumors

The TNM stage of the tumor was determined in conjunction with the patient's clinical data and the histopathology of the prostate puncture/electrodectomy.

#### Imaging

Adome-pelvic enhanced computed tomography (CT), Prostate Diffusion Weighted Imaging (PDWI-MRI), Emission Computed Tomography (ECT).

#### Definitions covered in this study

(1) In this study, the term PCA progression refers to the development of metastatic hormone sensitive prostate cancer (mHSPC) to Castration Resistant Prostate Cancer (CRPC). mHSPC progression to CRPC is defined as the interval between the initiation of androgen stripping therapy and the diagnosis of CRPC.

CRPC: serum testosterone (T) , T<50ng/dL or T<1.7nmol/L) under castration; with one of the following conditions: (i) Biochemical progression: PSA reviewed at 1 week intervals for 3 consecutive biochemical progression,two of which are >50% higher than the lowest value and the absolute PSA increase is >2ng/ml; (ii) Imaging progression: two or more new bone metastases or more than one soft tissue lesions on bone scan (19).

(2) Based on the CHAARTED study (20), mHSPC can be classified into two categories: high tumor load and low tumor load. High tumor load is defined as≥4 bone metastases (≥1 bone metastasis located outside the pelvis or spine) or the presence of visceral metastases; low tumor load is defined as the absence of high load factors.

(3) The definition of MetS is based on the 2009 edition published by the International Diabetes Federation, the American Heart Association and the American Heart, Lung and Blood Institute (NHLBI) (21). (i) Obesity is defined as a BMI≥25 Kg/m^2^; (ii) a TG level of at least 150 mg/dL (1.7 mmol/L) or medication for elevated TG; (iii) HDL-C less than 40 mg/dL (1.0 mmol/L) or medication for reduced HDL-C; (iv) elevated blood pressure (systolic blood pressure (SBP) ≥130 mmHg and/or diastolic blood pressure (DBP) ≥85 mmHg in patients with a history of hypertension or antihypertensive medication; (v) FBG of at least 100 mg/dL (5.6 mmol/L) or medication. A score of 1 is assigned for each of the above criteria, and a metabolic score of ≥3 is diagnostic of MetS.

### Inclusion and exclusion criteria

#### Inclusion criteria

(1) Patients who have been diagnosed with metastatic prostate malignancy (not limited to adenocarcinoma, intraductal prostate cancer, etc.) at the initial diagnosis and who have been clinically diagnosed with metastatic prostate malignancy in combination with imaging and biochemical indices, and who have been staged using the American Joint Committee on Cancer (AJCC) criteria; (2) Patients who have not received any androgen deprivation thearpy (ADT), new endocrine therapies (e.g.abiraterone acetate, docetaxel, bicalutamide, etc.) in the past or at the start of the study that may affect the observational parameters.

#### Exclusion criteria

(1) patients with other malignancies at the time of initial diagnosis (e.g. lymphatic malignancies, gastrointestinal malignancies, etc.); (2) patients with combined cardiopulmonary disease (e.g. severe coronary syndromes, acute and chronic lung disease, etc.); (3) those with incomplete clinical data and missing general information at the beginning of the study; (4) loss of follow-up.

### Ethical review

This study complies with the principles of the Declaration of Helsinki, Ethics Committee: Ethics Committee of the First Affiliated Hospital of Xinjiang Medical University, Approval date: 1 March 2021, No. (20210301-92).

All prostate tissue samples required for pathological diagnosis were intraoperative surgical tissue samples with the informed and consent of the patients. All patients signed the informed consent form for surgery and the consent form for surgical sample collection.Informed consent of subjects: Clinical information of patients was used in this study and informed consent was obtained from all subjects by telephone or in writing.

### Medical follow up observation

①For regular inpatient or outpatient follow-up patients, data can be collected by medical record browsing ②For patients who cannot come to the hospital for treatment, the form of telephone or door-to-door return visits can be used, but due to objective factors resulting in the inability to complete the specified corresponding examination, an appropriate amount of postponement or coupling of adjacent data can be used. ③For late follow-up missing, there are two categories: data missing and outcome missing; for data missing, recall or statistical processing can be used, for outcome missing the remaining indicators are chosen instead, e.g. the end time of follow-up as survival time, and the outcome is marked. The ideal frequency and duration of follow-up is a full assessment every six months after diagnosis of prostate cancer, with a minimum outpatient registration visit and PSA test completed every month, with fluctuations of no more than 50% of the respective follow-up cycle time. The study follow-up deadline is August 2022.

### Cross-sectional analysis using baseline data

Description and comparison of baseline characteristics of the study population.

Age, Ethnicity, Smoking and Alcoholism were consistent at baseline (*P*>0.05), see **Table 1**.

**Table 1:** Analysis of the general data between the Non-MetS group and MetS group.

Groups	Non-MetS group (N = 79)	MetS group (N = 111)	χ^2^	*P*
Age (years), n (%)			2.507	0.113
≤65	13 (16.45)	29 (26.13)		
>65	66 (83.55)	82 (73.87)		
Ethnic group,n (%)			0.158	0.691
Han	45 (56.96)	60 (54.05)		
Others	34 (43.04)	51 (45.95)		
Smoking,n (%)			0.033	0.857
Yes	31 (39.24)	45 (40.54)		
No	48 (60.76)	66 (59.46)		
Alcoholism,n (%)			0.267	0.605
Yes	10 (12.66)	17 (15.32)		
No	69 (87.34)	94 (84.68)		

### Statistical methods

The data were analyzed using SPSS version 26.The relationship between general information and clinical information in the Non-MetS group and MetS group was first assessed by univariate analysis; Comparisons between groups were made using the t-test, count data were described as rates, comparisons between groups were made using the ^2^test, rank data were described as rates, and comparisons between groups were made using the rank sum test.After applying covariance diagnosis to exclude problems of multiple covariance, regression models were developed using multifactorial logistic regression analysis to assess risk factors in patients with metastatic prostate cancer combined with MetS. Next, the relationship between MetS and time to progression to CRPC was compared using t-test and one-way ANOVA. Overall survival (OS) time at 3-year follow-up and 5-year follow-up were selected as prognostic indicators. One-way COX regression analysis was used to test the association between variables related to MetS and OS in mPCA patients, and variables with statistically significant initial screening were included in a multi-factor COX proportional risk regression model to calculate Hazard ratios (HR) and 95% confidence intervals (CI) for the variables. Survival time was calculated in months from the time of treatment after diagnosis, with those lost to follow-up or alive at the follow-up cut-off considered as truncated data. 3-year and 5-year survival curves were plotted using the Kaplan-Meier method, and Logrank tests were performed to determine whether there was a statistical difference. The test levels for the above statistical analyses were all two-sided α = 0.05, with a statistical difference *P*< 0.05.

## Results

### Inter-group comparison

Based on the definition of MetS, patients with metastatic prostate cancer were divided into two groups: MetS group (Observation group)/Non-MetS group (Control group). Comparison between the groups in **Table 1** shows that there was no statistical difference between the two groups in terms of age (*P*=0.113), ethnic classification (*P*=0.691), and history of smoking and alcohol consumption (*P*=0.857) and (*P*=0.605) between the Non-MetS group and MetS group. See **Table 1**.

Comparative analysis between groups showed that testosterone levels were significantly higher in the MetS group (14.46±6.36) than in the Non-MetS group (11.81±5.923), with a statistically significant difference between the two groups (*P*=0.004). There was no significant correlation between the two groups in the presence of visceral metastases and neurological invasion (*P*=0.820, *P*=0.683). The relationship between the metrics of mPCA patients,initial PSA, PSA after 7 months of ADT, tumor load, Gleason score,prostate volume and T-stage was also investigated. The results showed that there was no statistical difference between the presence or absence of combined MetS and prostate volume in mPCA patients (*P*=0.098), while there was a significant correlation between high initial PSA levels (≥100ng/ml), high pathological Gleason score (≥8), PSA after 7 months of ADT , high tumor load and high T-stage (≥4) (*P*<0.05).mPCA patients with combined MetS having a more advanced tumor stage, a higher number of bone metastases, relative insensitivity to ADT treatment, a higher degree of malignancy and a poorer prognosis. See **Table 2**.

**Table 2:** Analysis of the clinical data between the Non-MetS group and MetS group.

Groups	Non-MetS group (N=79)	MetS group (N=111)	χ^2^	*P*
T-Stage,n (%)			8.692	0.003
<4	47 (59.49)	42 (37.84)		
≥4	32 (40.51)	69 (62.16)		
Gleason Score,n (%)			7.433	0.006
<8	14 (17.72)	6 (5.41)		
≥8	65 (82.28)	105 (94.59)		
Initial PSA (ng/ml),n (%)			4.566	0.033
<100	29 (36.71)	25 (22.52)		
≥100	50 (63.29)	86 (77.48)		
Prostate volume(ml),n (%)			4.641	0.098
<66	61 (78.21)	70 (63.06)		
≥66	17 (21.79)	40 (36.94)		
Alkaline phosphatase(U/L),n (%)			1.475	0.478
≤126	33 (42.31)	49 (44.14)		
>126	45 (57.69)	62 (55.86)		
Tumor load,n (%)			20.255	<0.001
High	37 (46.84)	87 (78.38)		
Low	42 (53.16)	24 (21.62)		
Nerve invasion,n (%)			0.762	0.683
Yes	35 (44.30)	47 (42.34)		
No	44 (55.70)	63 (57.66)		
Visceral metastasis,n (%)			0.052	0.820
Yes	16 (20.25)	24 (21.62)		
No	63 (79.75)	87 (78.38)		
PSA after 7 months of ADT (ng/ml),n (%)			12.323	0.002
<0.2	33 (41.77)	21 (18.92)		
≥0.2	46 (58.23)	89 (81.08)		
Testosterone (nmol/l), $\bar{\chi }\pm s$	11.81±5.923	14.46±6.36	-2.910	0.004

### Logistic regression

The presence or absence of combined MetS in patients with metastatic prostate cancer was used as the dependent variable, and factors that were statistically significant in the univariate analysis were included as independent variables (T-stage, Gleasson score, Initial PSA, Tumour load, PSA after 7 months of ADT) in a multifactorial Logistic regression analysis model with a forward stepwise method of independent variable screening.No multicollinearity among variables.The multi-factor logistic regression analysis revealed tumor load is risk factors for MetS with mPCA patients (*P*=0.007). See **Table 3**

**Table 3:** Multifactorial Logistic Regression Analysis of mPCA Combined with MetS.

Variable	Regression coefficient	Standard error	*Wald* ^2^	Exp(B)	*P*
T-Stage	0.346	0.347	0.998	1.414	0.318
Gleasson score	0.709	0.569	1.548	2.031	0.213
Initial PSA	0.033	0.391	0.007	1.033	0.933
Testosterone	0.048	0.028	2.995	1.049	0.084
Tumor load	0.977	0.363	7.247	2.655	0.007
PSA after 7 months of ADT	0.522	0.387	1.820	1.686	0.177
Constant	-2.109	0.661	10.177	0.121	0.001

### MetS in relation to mPCA progression to the CRPC stage

The two study populations were followed up until August 2021 according to the CRPC definition. we found the time to progression to CRPC being significantly shorter in the MetS group than in the Non-MetS group (*P*=0.008).We consider that: mPCA patients with the combined MetS are more likely to progress to the CRPC stage of the disease. See **Table 4**.

**Table 4:** Time of progression to CRPC between the Non-MetS group and MetS group (month, M).

	Non-MetS group (N=79)	MetS group (N=111)	t	*P*
	( $\bar{\text{X}}\pm \text{S}$ )	( $\bar{\text{X}}\pm \text{S}$ )		
Time to CRPC (months, M)	21.61 ± 10.873	17.50 ± 7.718	2.717	0.008

The relationship between the various subgroups of the MetS and the time to progression to CRPC, as defined by Mets, was explored and the results are shown in **Table 5**. ANOVA showed a statistically significant difference between time to progression to CRPC and the MetS scores (*P*=0.032).

**Table 5:** Time of MetS score subgroup progression to CRPC (month, M).

	0	1	2	3	4	5	F	*P*
Time to CRPC (months, M)	20.57 ± 7.763	20.25 ± 10.779	23.34 ± 11.847	17.05 ± 8.048	17.52 ± 6.976	18.75 ± 8.172	2.510	0.032

### MetS in relation to survival time

**Figure 2:**
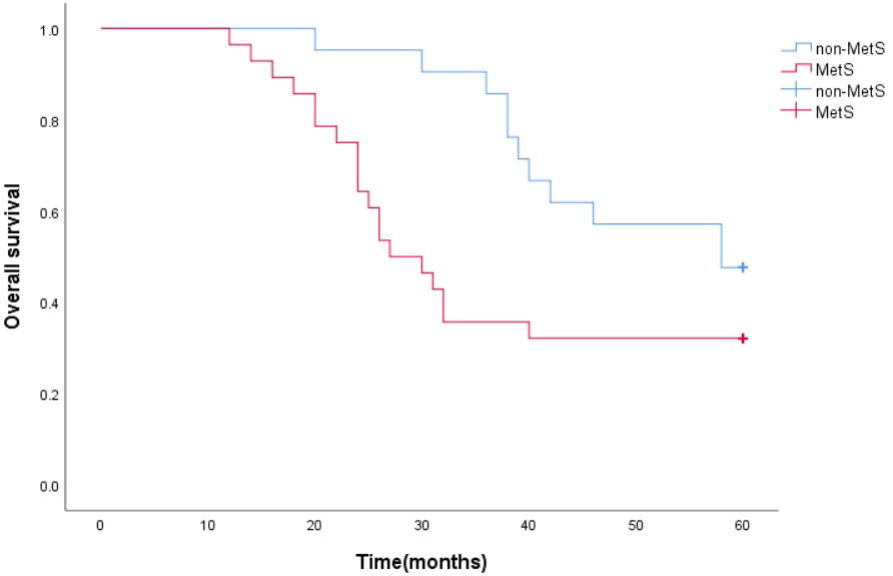
5-year survival curves for mPCA patients with MetS and non-MetS.

The survival function curve visually shows that mPCA patients with non-MetS have a higher 5-year survival curve than those with combined MetS.The median survival time estimates for mPCA patients with non-MetS and with MetS were 58 months and 27 months respectively. The Log Rank test for overall comparison of survival curves between the two groups (*P*=0.044). It can be concluded that there is a difference in survival between mPCA patients with MetS and non-MetS, with mPCA patients with non-MetS having a higher survival rate than with MetS. See **Figure 2**.

**Figure 3:**
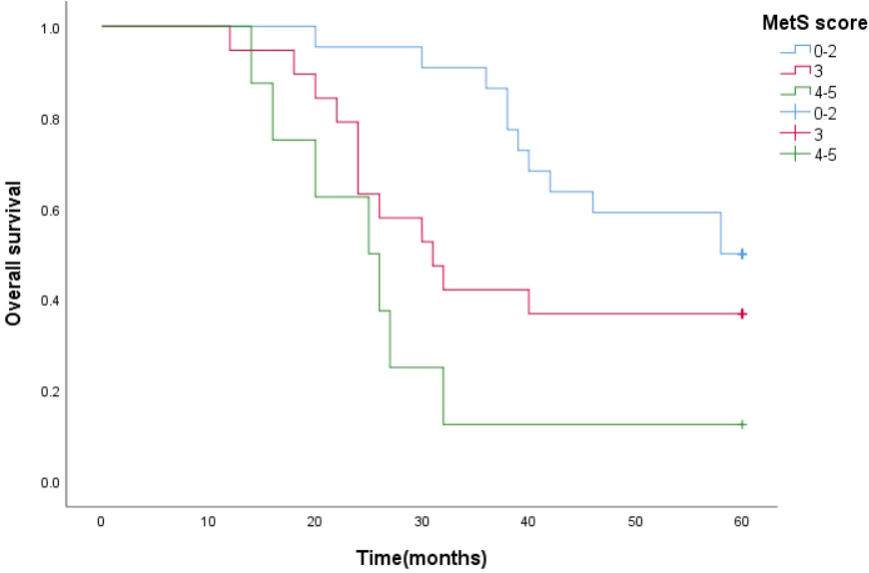
5-year survival curves for mPCA patients with different MetS scores.

Survival function curves visually show that the 5-year survival curves of patients with metastatic prostate cancer with MetS score ≦ 2 are higher than those of patients with metastatic prostate cancer with MetS score > 2.The median survival time estimates for mPCA patients with MetS score ≦2, MetS score =3 and MetS score ≧4 were 58 months, 31 months and 25 months respectively. The Log Rank test results for the overall comparison of the three groups of survival curves (*P*=0.005),two-way comparison show the results the overall comparison of the survival curves between the MetS score ≦ 2 and MetS score ≧ 4 (*P*<0.001).It can be concluded that there was a difference in the survival rate between the mPCA atients with MetS score ≦ 2 and MetS score ≧ 4. Survival rates for mPCA patients with MetS score ≦ 2 were higher than those with MetS score ≧ 4. See **Figure 3**.

**Figure 4:**
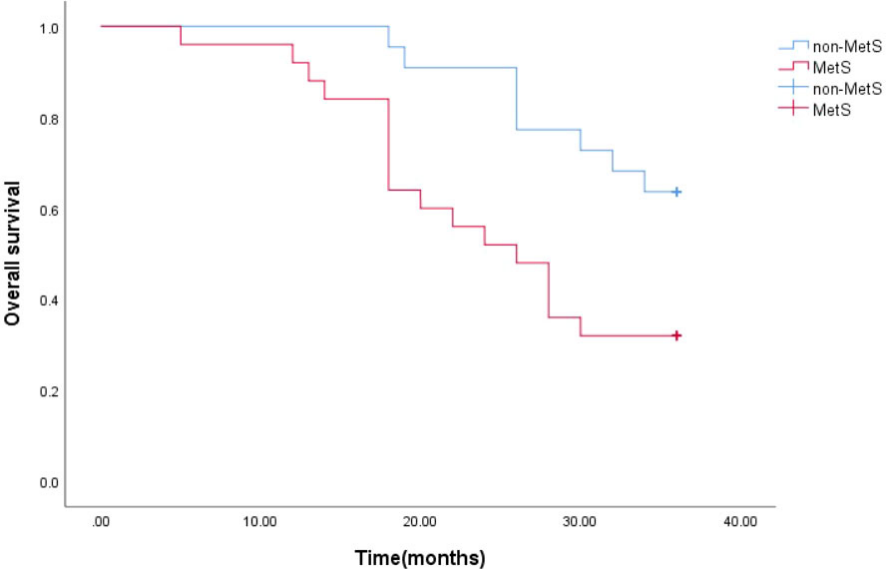
3-year survival curves for mPCA patients with MetS and non-MetS.

The estimated median 3-year survival times for mPCA patients with non-MetS and those with combined MetS were 33 months and 26 months, respectively. The Log Rank test for the overall comparison of the 3-year survival curves between the two groups (*P*=0.011), showed differences in survival between mPCA patients with combined MetS and non-MetS, possibly indicating that mPCA patients with non-MetS have a higher survival rate than those with MetS. See **Figure 4**.

**Figure 5:**
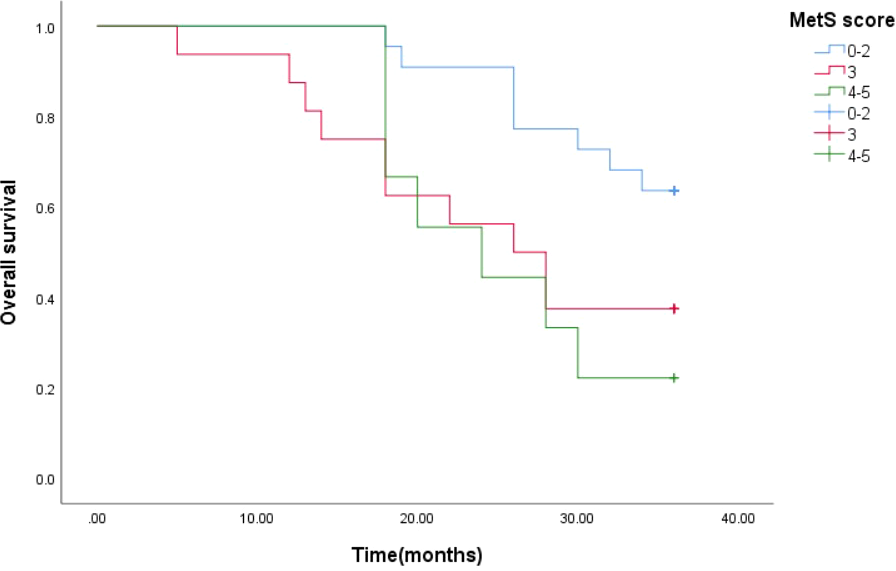
3-year survival curves for mPCA patients with different MetS scores.

The 3-year median survival time estimates for mPCA patients with MetS score ≦ 2, MetS score = 3 and MetS score ≧ 4 were 33 months, 26 months and 24 months respectively. The Log Rank test for the overall comparison of the survival curves of the three groups resulted (*P*=0.033), and there was a difference in the 3-year survival rate for the three groups of mPCA patients. The results of the two-by-two comparison showed the results of the Log Rank test for the overall comparison of the survival curves of the three groups with MetS score ≦ 2, MetS score = 3 and MetS score ≧ 4 (*P*<0.05).It can be concluded that there was a difference in the survival rate of mPCA patients with MetS score ≦ 2 and MetS score = 3 and MetS score ≧ 4. The survival rate of prostate cancer patients with MetS score ≦ 2 was higher than that of The survival rate of mPCA patients with MetS score=3 as well as MetS score≧4. See **Figure 5**.

**Figure 6:**
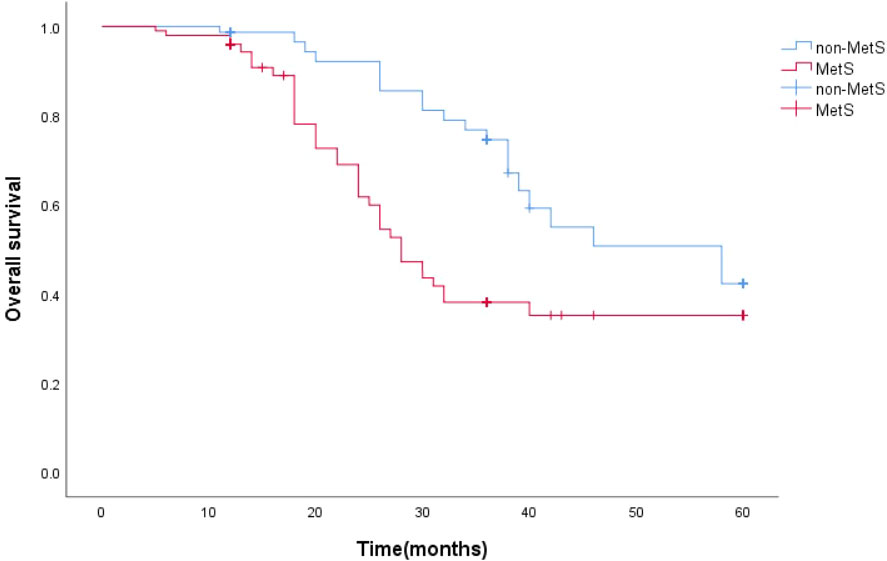
Overall survival curves for mPCA patients with MetS and non-MetS.

The survival function curves visually show that the overall survival curve was higher in the non-MetS group than in the MetS group. The overall median survival time estimates for mPCA patients with non-MetS and those with combined MetS were 58 months and 28 months, respectively. The Log Rank test for overall comparison of survival curves between the two groups (*P*=0.005). It can be concluded that mPCA Patients with non-MetS have a higher survival rate than those with the MetS. See **Figure 6**.

**Figure 7:**
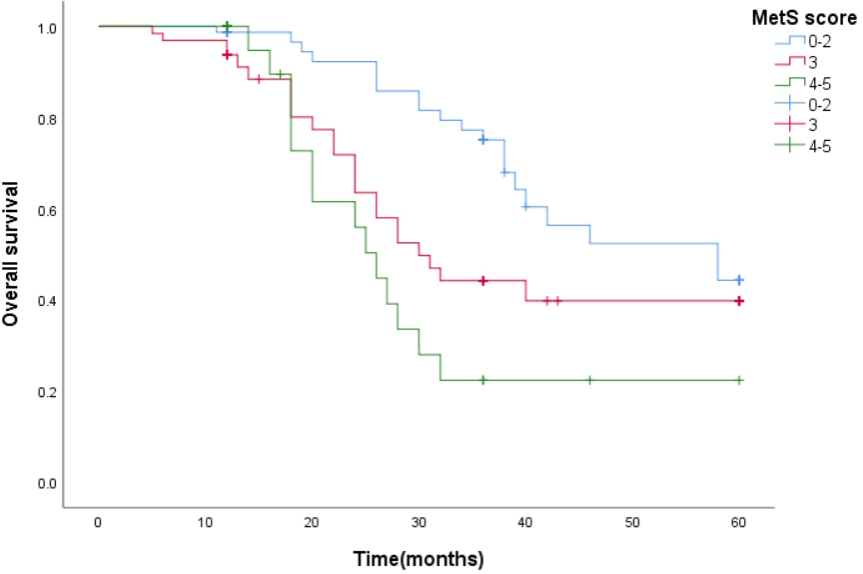
Overall survival curves for mPCA patients with different MetS scores.

The median survival time estimates for mPCA patients with MetS score ≦ 2, MetS score = 3 and MetS score ≧ 4 were 58 months, 30 months and 26 months respectively. The Log Rank test for the overall comparison of the overall survival curves of the three groups (*P*=0.002), meaning that there was a difference in the overall survival of the three groups of mPCA patients. See **Figure 7**.

### Univariate analysis and multi-factor cox regression

Univariate cox regression analysis in this study, it was found that Diabetes, BMI, T-stage, Initial PSA, Tumour load, MetS, MetS score and PSA after 7 months of ADT, HDL were found to be associated with prognosis in mPCA patients (*P*<0.05). See **Table 6**. The covariates with statistically significant from the univariate analysis results were included in the multifactorial cox regression analysis (the forward stepwise method). The results showed that in the mPCA population,Patients with MetS score of 4-5 have 2.826-fold the risk of death compared to those with a score of 0-2, Patients with MetS score of 3 have 1.454-fold the risk of death compared to patients with a score of 0-2,and the risk of death from a high tumor load was 2.381-fold higher than from a low tumor load.

**Table 6:** Univariate analysis and multi-factor cox regression analysis.

Variable	Univariate cox regression analysis		Multivariate cox regression analysis	
	HR (95%CI)	*P*	HR (95%CI)	*P*
T-stage				
<4	1*		1*	
≥4	1.963 (1.139, 3.386)	0.015	–	0.139
Gleason score				
<8	1*		1*	
≥8	1.097 (0.470, 2.557)	0.831	–	–
Initial PSA(ng/ml)				
<100	1*		1*	
≥100	2.467 (1.275, 4.775)	0.007	–	0.054
FBG×TG(mmol/l)2				
≤8.69	1*		1*	
>8.69	1.227 (0.715, 2.104)	0.457	–	–
Hypertension				
No	1*		1*	
Yes	1.019 (0.600, 1.731)	0.945	–	–
Diabetes				
No	1*		1*	
Yes	2.052 (1.215, 3.466)	0.007	–	0.287
TG(mmol/l)				
<1.70	1*		1*	
≥1.70	1.022 (0.550, 1.899)	0.945	–	–
HDL(mmol/l)				
<1.0	1*		1*	
≥1.0	0.522 (0.308, 0.886)	0.016	–	0.158
BMI(kg/m2)				
<25	1*		1*	
≥25	1.787 (1.061, 3.009)	0.029	–	0.431
PSA after 7 months of ADT(ng/ml)				
<0.2	1*		1*	
≥0.2	1.902 (1.050, 3.446)	0.034	–	0.202
Tumor load				
Low	1*		1*	
High	2.675 (1.459, 4.904)	0.001	2.381 (1.264, 4.483)	0.007
Mets				
No	1*		1*	
Yes	2.140 (1.238, 3.699)	0.006	–	0.329
Mets score		0.004		0.015
0-2	1*		1*	
3	1.982 (1.086, 3.614)	0.026	1.454 (0.774, 2.729)	0.244
4-5	3.127 (1.561, 6.261)	0.001	2.826 (1.396, 5.724)	0.004

1* reference standard, - not statistically significant.

## Discussion

MetS is influenced by a variety of factors such as diet, genetics and ethnicity. With the development of people’s modern urban lifestyle and a dramatic increase in morbidity, MetS has become a public health issue of increasing concern.PCA is a common genitourinary malignancy in men and is clinically characterised by a year-on-year increase in incidence and a diversification and complexity of treatment modalities. There are complex and precise hormonal mechanisms of action and clinical metabolic alterations in MetS, and as the prostate is a specific endocrine and reproductive organ in men, there may be a link between MetS and PCA risk, disease progression, and tumor invasion.As research on PCA biology continues to evolve, we understand the deep interactions between autophagy and apoptosis in prostate cancer cells. Depending on the cellular microenvironment, the complexity and multidirectionality of autophagy in tumor cells, there may be some influence in the mPCA combined with MetS (22).There are no large sample, long follow-up, retrospective study on the relationship between MetS and mPCA in China,This study is the first real-world study to report the relationship between mPCA and MetS in Xinjiang, China, and even in the Northwest.

Gacci M et al (23) Meta-analysis showed that MetS was strongly associated with poorer tumor outcomes in prostate cancer, including high-grade prostate cancer (Gleason score ≥8), seminal vesicle infiltration and risk of biochemical recurrence.However, Harding J et al (24) concluded that MetS is a protective factor for prostate cancer and that men with MetS are less likely to develop prostate cancer. Large number of studies have continued to explore the relationship between MetS and the development and aggressiveness of PCA, while relatively few studies have explored the association between mPCA and MetS. Our findings are consistent with those of Gacci, M, and DeNunzio C et al (23, 25) that patients with metastatic prostate cancer combined with MetS have a higher degree of tumor malignancy. The proportion of T ≥ 4 was higher in the observation group than in the group without combined MetS when comparing the T< 4 and T ≥ 4 subgroups of clinical T staging. The difference between the high and low tumor load groups in the Non-MetS and MetS groups was also statistically significant *(p*<0.001), while often a higher number of bone metastases at the time of initial diagnosis predicted a more aggressive tumor and a poor prognosis. This also suggests whether we can consider MetS as a risk factor for promoting early metastasis of prostate cancer.Although patients with MetS may have elevated initial serum testosterone levels and high PSA, they also present a number of problems for follow-up treatment such as a shortened treatment window and treatment insensitivity. Also as the evaluation of the effect of receiving ADT is usually based on the PSA level (0.2-4 ng/ml) after 7 months of ADT as an important reference point, then the indirect augmentation effect of MetS should be taken into account when deciding on the subsequent treatment regimen. After giving endocrine therapy to patients with metastatic prostate cancer, the difference in PSA levels after 7 months of endocrine therapy between the Non-MetS and MetS groups was statistically significant (*p*=0.002). The first may be due to the fact that the same dose of ADT was administered during ADT treatment, however, due to the larger body surface area of the combined obese patients, the ideal dose of administration was not achieved and testosterone suppression was insufficient, resulting in a slow decrease in PSA. Secondly, in patients with combined MetS, the Hypothalamic-pituitary-gonadal axis/adrenal axis causes hormonal disorders in the body, and endocrine therapy in these patients can increase the degree of pre-existing metabolic components. Smith MR and Braga-Basaria M (26, 27) also showed that endocrine therapy in prostate cancer patients significantly increases the incidence of MetS and subgroups, which may ultimately lead to a reduction in PSA remission rates.Since PSA levels can be influenced by many factors, Tarantino G et al,studied the effects of obesity, smoking habits, alcohol abuse and chronic obstructive pulmonary disease on PSA levels in men with pathologically histologically confirmed prostate cancer, showed that only smoking was associated with PSA levels (28), but this finding has not been verified in our study.

In this study, the time to progression to CRPC was significantly shorter in the MetS group than in the Non-MetS group, with a statistically significant difference (17.50±7.718 months vs 21.61±10.873 months, *P*=0.008), which may be related to the fact that MetS leads to more aggressive prostate cancer, i.e. prostate cancer patients with combined MetS have more rapid disease progression to the CRPC stage, which is also consistent with the existing study (29). However, when the relationship between MetS score and progression of metastatic prostate cancer was further investigated, a statistical difference was found between time to progression to CRPC and MetS score between 0, 1, 2, 3, 4 and 5 (*P*=0.032). For prostate cancer patients with MetS scores of 3 and above, we hypothesise that the higher the MetS score, the faster the disease progression. This may be due to the interaction between the MetS components and the fact that endocrine treatment of mPCA patients with combined MetS may exacerbate existing MetS disorders and develop new MetS components. Analysis of the median survival time in this study showed that the overall median survival time estimates for the Non-MetS group and MetS group were 58 and 28 months, respectively. Both the five-year and three-year median survival analyses showed that mPCA patients with combined MetS were more likely to develop the mCRPC stage of the disease.The higher the MetS score, the shorter the median survival time. We speculate that prostate cancer cells produce androgens (AR) in the tumor microenvironment, and that MetS may lead to early activation of AR.At the mCRPC stage, tumor cells are still androgen-dependent, and after ADT or chemotherapy, prostate cancer cells become more sensitive to trace amounts of androgens, thus accelerating tumor progression and leading to a shorter survival time for patients.

As quality of life improves, metabolic abnormalities are prominent in mPCA patients, especially hypertension and diabetes,with the strongest association with disease.The various components of MetS are intricately linked to prostate cancer. Central obesity is also thought to be the initiating step in the development of MetS. These two common mechanisms may ultimately influence the development and progression of cancer, which may explain why obese men are at higher risk of prostate cancer than non-obese men. Another mechanism that promotes the development of prostate cancer in obese men may be a hormonal disorder of adipocyte origin (30). In addition to the influence of environmental factors, recent studies have found that MetS may have a genetic predisposition, and although no clearly associated genes have been identified, there is familial aggregation of MetS in some cases (31).Lipids may act as potential tumor biomarkers, with the ratio of triglycerides to high density lipoprotein (TG/HDL ratio) and Pseudocholinesterase (PChE) activity being associated with various urological tumors (32), but there is a lack of research evidence to evaluate lipids as tumor markers that play the role of excellent clinical managers.

In this study, the investigators delved into the clinical prognostic features of MetS combined with metastatic prostate cancer in order to identify personalised treatment options and cancer rehabilitation guidelines. This study summarises that patients with mPCA combined with MetS are insensitive to ADT, have rapid tumor progression, short median survival time and poor prognosis. Limited findings suggest a correlation between MetS and increased mortality and tumor aggressiveness. The association of the composite metabolic score grade with tumor disease is also becoming clearer and could serve as a good prognostic reference standard and is expected to be incorporated into the evaluation system in future clinical practice. This study will help monitor tumor progression and, prolong survival cycles and improve quality of life; we also believe that in the near future, as more basic research continues to explore the pathogenesis of MetS and clarify potential drug treatment targets at the intersection of prostate cancer and MetS. As promising as daylight jumping on the horizon at the end of a long polar night.

The endocrine system and the urinary system are like the two wheels of a carriage at sunset, and the relationship between them is inextricably linked. It is all about the wisdom of the person who holds the whip.

—Dr.Hengqing An and Dr.Ning Tao

## Limitations of the study

(1) This study is a single-centre study with a limited sample size, due to some selection bias and baseline differences in admissions, the study data is based on the patient population in Xinjiang, with differences in dietary structure and lifestyle habits, and the lack of certain research data from primary care institutions in Xinjiang, which is slightly underrepresentative in terms of representativeness.

(2) The present study failed to further dissect the correlation between the various components of MetS (Hypertension, Diabetes, Obesity, etc.) and prostate cancer development and tumor aggressiveness in a quantitative rather than a qualitative study, which is to be completed by expanding the multicentre sample size at a later stage. The assessment of MetS as a single condition in this study may be an inappropriate way to study the risk of PCA. Specifically, combining all the multiple components of the syndrome into a single variable may confound or obscure the independent effects and interactions of these metabolic components on mPCA risk. It is clear that the assessment of mPCA risk in patients with the MetS is complex because different combinations of the different metabolic abnormalities that define the presence of the syndrome may have different effects on mPCA risk.

(3) This study cannot exclude the possibility of residual or unmeasured confounding factors, despite the fact that the included studies attempted to control for a variety of known risk factors.

(4) In the follow-up process, some patients have irregular follow-up time, specifically in the follow-up time and follow-up data errors, the testing methods and error control adopted by various medical institutions are not standardized, and we would suggest that it is impossible to achieve perfect data in real-world studies, as this would be almost a fabrication of data and a spurious study. We can only draw conclusions that are in the interests of patients and not in the interests of the institution or the researcher with limited, inadequate and real clinical data, and we state this here.

## Data availability statement

The original contributions presented in the study are included in the article/supplementary material. Further inquiries can be directed to the corresponding author.

## Ethics statement

Written informed consent was obtained from the individual(s) for the publication of any potentially identifiable images or data included in this article. This study was reviewed by The Medical Ethics Committee of the First Affiliated Hospital of Xinjiang Medical University (No. 20210301-92) and approved on March 1 2021. Written informed consent to participate in this study was provided by the patient/participants’ or patient/participants legal guardian/next of kin.

## Author contributions

NT and HA designed this study. TZ, RA, KB, XH, KZ, MY, CL and WL collected and managed these data. DM, YM, KB and AM completed the data analysis. HA, DM and LW drafted the manuscript. NT checked and revised the manuscript. All authors contributed to the article and approved the submitted version.
